# Supplementary figures and images for: WRKY transcription factor family in lettuce plant (Lactuca sativa): Genome-wide characterization, chromosome location, phylogeny structures, and expression patterns
Source: PeerJ. 2022 Oct 18;10:e14136. doi: 10.7717/peerj.14136 (PMC9586095; doi:10.7717/peerj.14136)

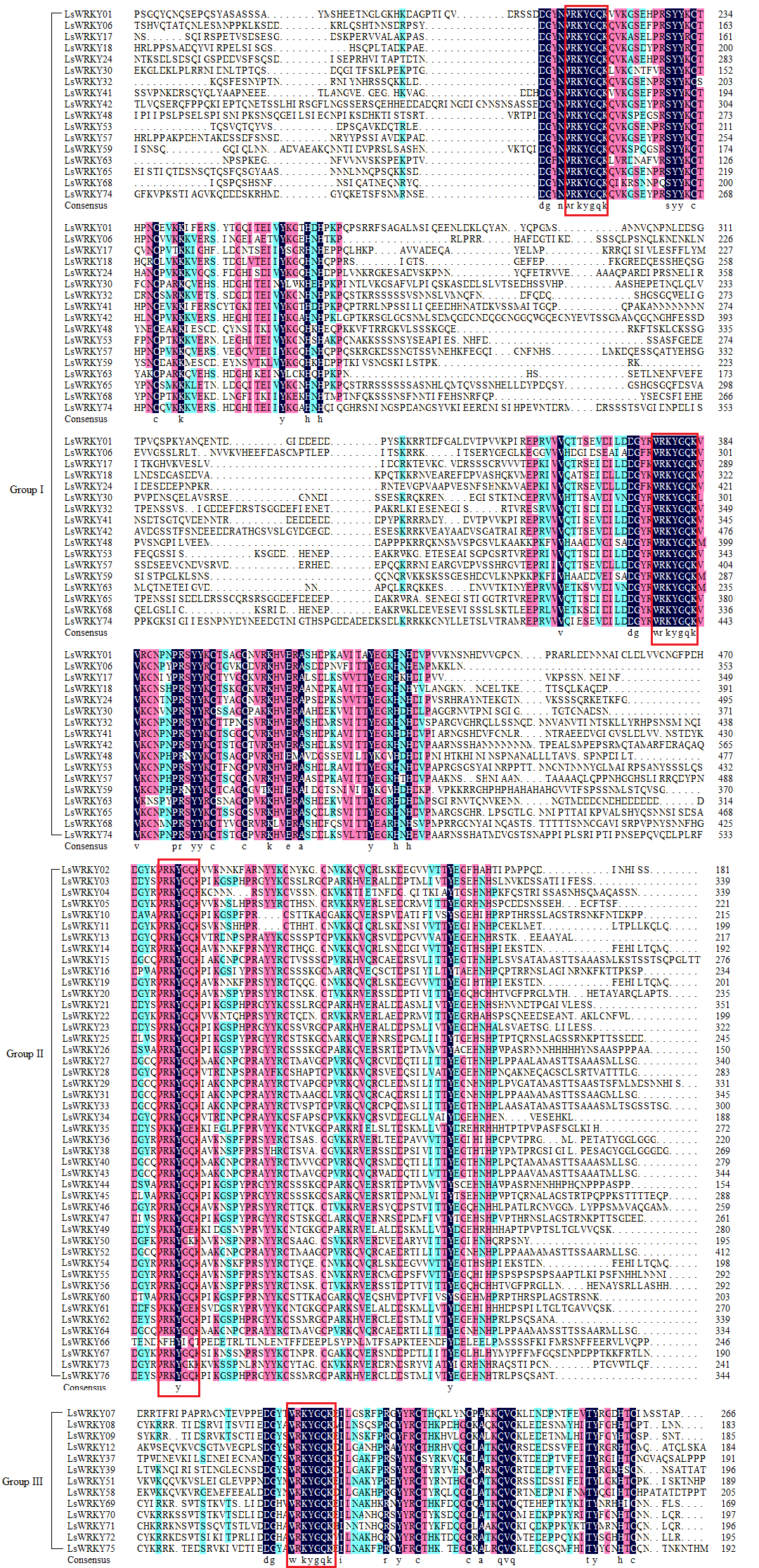

Supplement: Figure S1 — The red box represented the conservative domain WRKYGQK of WRKY TFs; amino acids of different colors indicated different degrees of similarity. [file peerj-10-14136-s001.png]

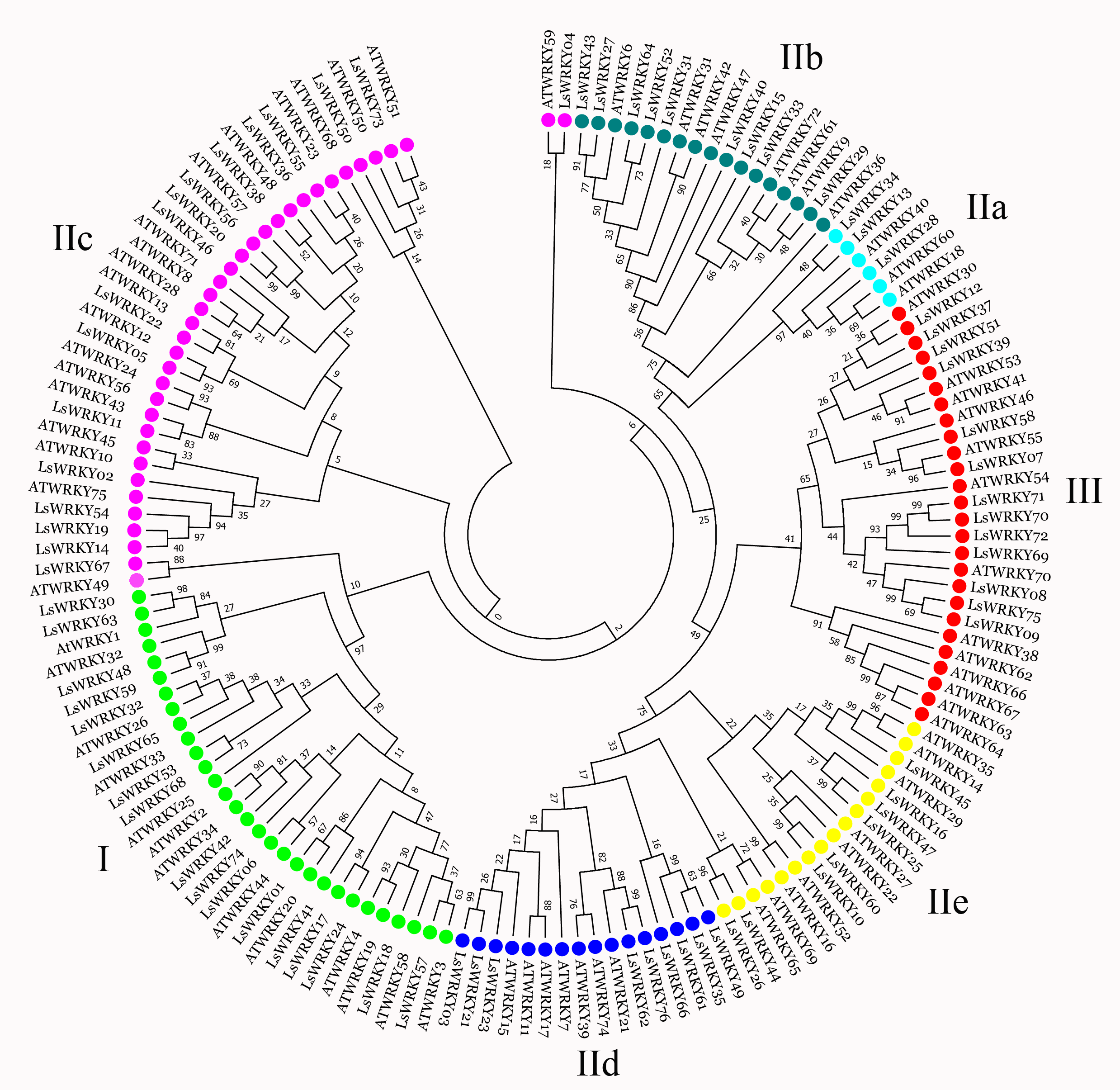

Supplement: Figure S2 — Different colored boxes represented different WRKY subfamily TFs. [file peerj-10-14136-s002.png]

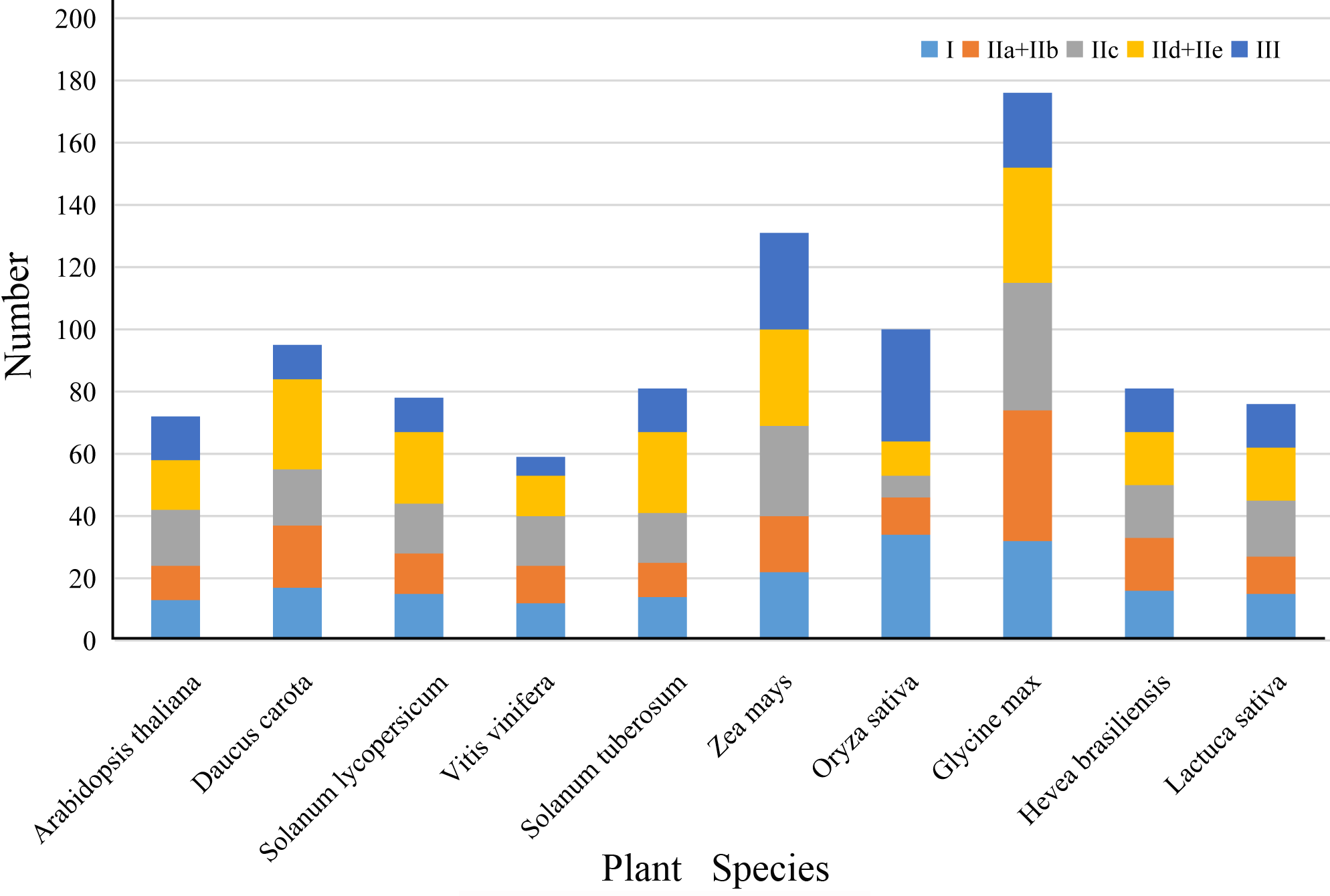

Supplement: Figure S3 — Different colors represent different subfamily WRKY TFs. [file peerj-10-14136-s003.png]
